# Influenza A–induced cystic fibrosis transmembrane conductance regulator dysfunction increases susceptibility to *Streptococcus pneumoniae*

**DOI:** 10.1172/jci.insight.170022

**Published:** 2023-07-24

**Authors:** Erin Y. Earnhardt, Jennifer L. Tipper, Adonis D’Mello, Ming-Yuan Jian, Elijah S. Conway, James A. Mobley, Carlos J. Orihuela, Hervé Tettelin, Kevin S. Harrod

**Affiliations:** 1 Department of Anesthesiology and Perioperative Medicine, Heersink School of Medicine, University of Alabama at Birmingham, Birmingham, Alabama, USA.; 2 Department of Microbiology and Immunology, Institute for Genome Sciences, University of Maryland School of Medicine, Baltimore, Maryland, USA.; 3 Department of Microbiology, Heersink School of Medicine, University of Alabama at Birmingham, Birmingham, Alabama, USA.

**Keywords:** Infectious disease, Virology, Bacterial infections, Influenza, Ion channels

## Abstract

Influenza A virus (IAV) infection is commonly complicated by secondary bacterial infections that lead to increased morbidity and mortality. Our recent work demonstrates that IAV disrupts airway homeostasis, leading to airway pathophysiology resembling cystic fibrosis disease through diminished cystic fibrosis transmembrane conductance regulator (CFTR) function. Here, we use human airway organotypic cultures to investigate how IAV alters the airway microenvironment to increase susceptibility to secondary infection with *Streptococcus pneumoniae* (*Spn*). We observed that IAV-induced CFTR dysfunction and airway surface liquid acidification is central to increasing susceptibility to *Spn*. Additionally, we observed that IAV induced profound transcriptional changes in the airway epithelium and proteomic changes in the airway surface liquid in both CFTR-dependent and -independent manners. These changes correspond to multiple diminished host defense pathways and altered airway epithelial function. Collectively, these findings highlight both the importance of CFTR function during infectious challenge and demonstrate a central role for the lung epithelium in secondary bacterial infections following IAV.

## Graphical abstract

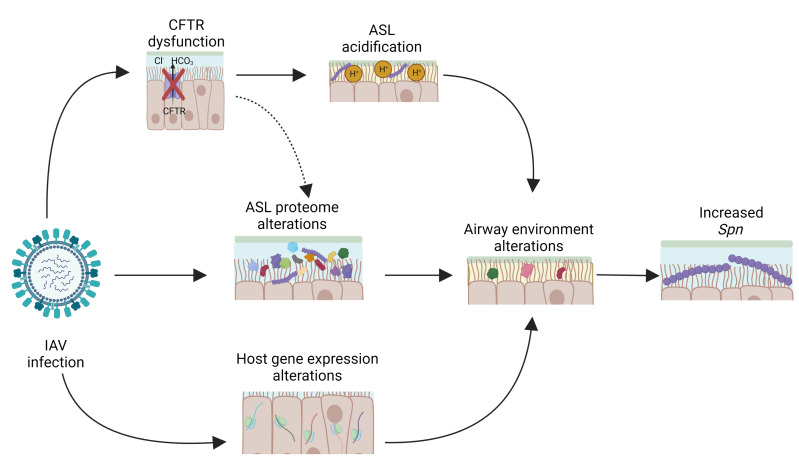

## Introduction

Respiratory viral infections are commonly complicated by secondary infection with bacterial pathogens, leading to an increase in the severity of the viral infection (1). *Streptococcus pneumoniae* (*Spn*), a Gram-positive bacterium and the leading cause of global community-acquired pneumonia (2), is a frequent cause of infection secondary to influenza A virus (IAV), increasing the morbidity and mortality of IAV (3, 4). This is particularly evident during IAV pandemics. In an analysis of archived samples from the 1918 IAV pandemic, bacterial infection secondary to IAV correlated with an increase in mortality, and *Spn* was found in 71% of cases of bacterial infections secondary to IAV (5, 6). More recently, in the 2009 H1N1 IAV pandemic, *Spn* was found in 35% of mild and severe IAV cases and was closely associated with increased morbidity and mortality (7).

Previous reports have suggested underlying causes of secondary bacterial infection with *Spn* following IAV. This includes a modulation of immune-mediated defense by IAV that leads to reduced bacterial clearance (8–11). Conversely, secondary infection with *Spn* has also been shown to interfere with the host immune response to IAV (12). Additionally, synergistic interactions between IAV and *Spn* neuraminidases have been shown to contribute to increased pathogenesis during coinfection, including evidence that the neuraminidase protein of IAV provides free sialic acids that serve as a nutrient source for *Spn* (13–15). IAV also increases nutrient availability to *Spn* in the airway by increasing capillary permeability, allowing for an influx of glucose and other metabolites into the lower airway (16). IAV and *Spn* have been shown increase *Spn* binding to respiratory epithelial cells through direct IAV-*Spn* interactions (17). IAV-*Spn* interactions have also been shown to alter the bacterial proteome in a host-independent manner (18), and bacterial genes that allow for increased growth of *Spn* in the altered airway environment caused by IAV have been identified (19). Finally, IAV reduces mucociliary clearance in the airway, leading to a reduction in clearance of *Spn* (20). Yet despite these reports, substantial gaps in our knowledge remain, particularly as it pertains to the role of the lung airway epithelium.

Host defense against bacterial pathogens in the airway is a delicate balance of hematopoietically derived immune responses and constitutive epithelium-derived defense mechanisms, such as mucociliary clearance and luminal antibacterial proteins. Many of these important epithelium-derived host defense mechanisms have been ascertained through the study of human genetic diseases that increase susceptibility to bacterial infections. One of the most common genetic disorders affecting host defense in the airway is cystic fibrosis (CF) resulting from mutations in the CF transmembrane conductance regulator (CFTR) chloride and bicarbonate ion channel (21). Loss of CFTR function leads to an increase in inflammation (22) and a dysregulation of the airway microbiota (23). CFTR dysfunction is linked to disruptions in the airway epithelium function (24) and changes in the airway proteome, with many changes involving pathways that are essential for host defense in the airway (25). Additionally, CFTR dysfunction causes an acidification of the airway surface liquid (ASL) through its bicarbonate channel activity (26), leading to decreased pH-dependent host defense mechanisms in the airway lumen (27–29). Collectively, clinical and experimental findings suggest CFTR function is a critical mediator of bacterial host defense in the human airway.

Here, we utilize a differentiated organotypic model of primary human airway epithelial cells derived from various human anatomical pulmonary regions to elucidate the role of the lung epithelium in the development of bacterial infections secondary to IAV infection. We report that IAV-induced CFTR dysfunction and subsequent ASL acidification increases the bacterial burden in organotypic human airway cultures against a framework of both CFTR-dependent and CFTR-independent mechanisms, illustrating a central role for the airway epithelium in the development of secondary bacterial infections after IAV.

## Results

### IAV increases susceptibility to Spn in the airway epithelium.

**Figure 1:**
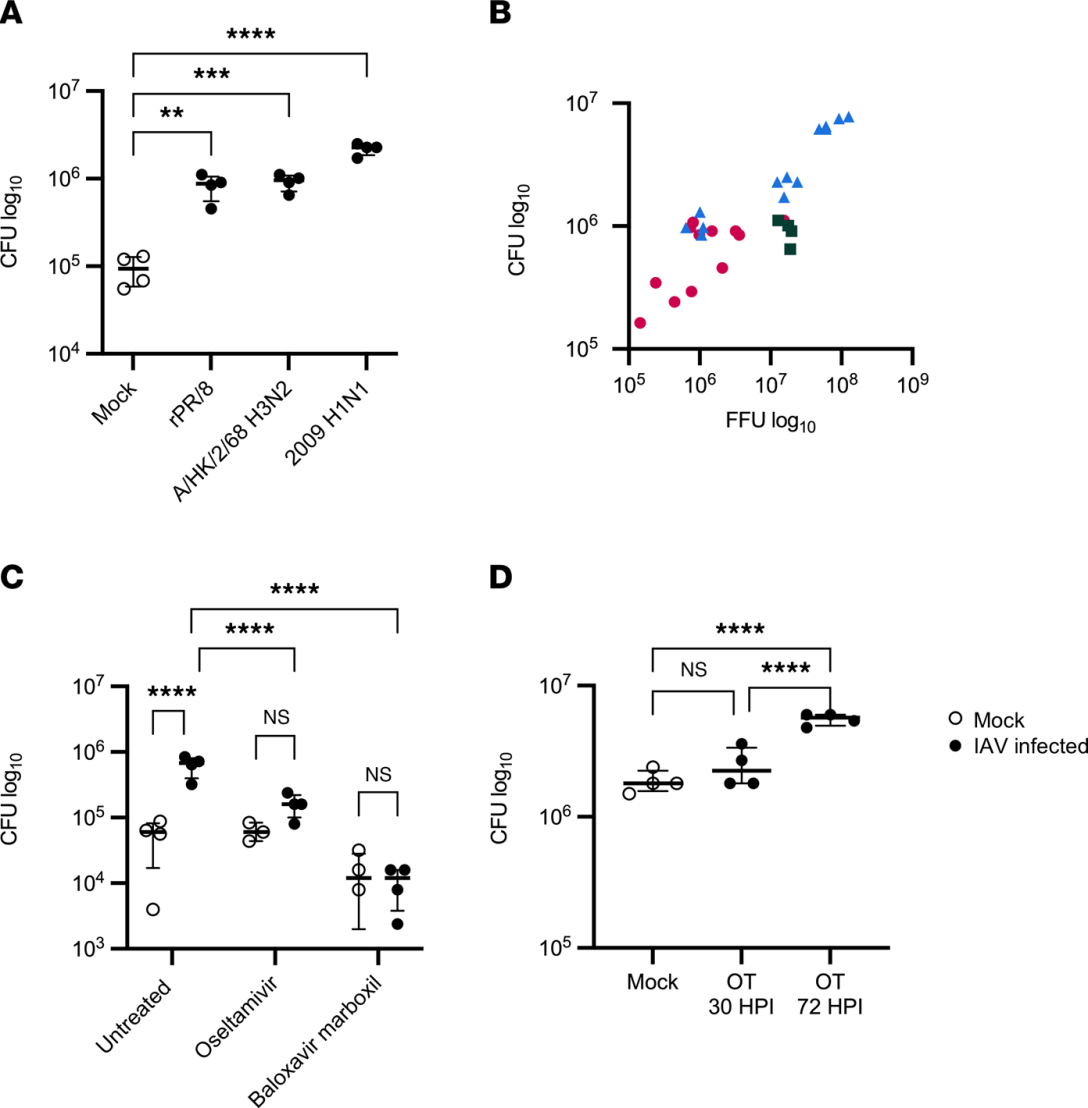
IAV replication increases *Spn* in the airway. (**A** and **B**) HBECs were infected with 150,000 PFU of IAV for 72 hours, followed by infection with 1,000 CFU of *Spn* (*n* = 4–16). (**C**) HBECs were infected with 100,000 PFU of IAV (pH1N1) and treated basally with 1 mM oseltamivir or 100 nM baloxavir marboxil for 72 hours, followed by infection with 1,000 CFU of *Spn* (*n* = 3–4). (**D**) HBECs were infected with 100,000 PFU of IAV (pH1N1) and then treated basally with 1 mM oseltamivir either 30 or 72 hours after IAV infection. After 72 hours of IAV infection, HBECs were apically infected with 1,000 CFU of *Spn*. *Spn* was quantified by vertical plating of apical washes collected after 6 hours of *Spn* infection (*n* = 4). All panels except **C** were analyzed by 1-way ANOVA with Dunnett’s (**A**) or Tukey’s (**C** and **D**) multiple-comparison test. **P < 0.01; ***P < 0.001; ****P < 0.0001. Brackets indicate median and interquartile range. Panel **C** was analyzed by Pearson’s correlation: *r* = 0.9348, 95% CI = 0.8645 to 0.9692, *P* < 0.0001. Open circles indicate mock IAV infection; closed circles indicate IAV infection. In panel **B**, magenta circles indicate rPR/8, green squares indicate H3N2, and blue triangles indicate pH1N1.

To establish the relevance of our model for the study of secondary bacterial infections after IAV, primary human bronchial epithelial cells (HBECs) from multiple donors were propagated and cultured on air-liquid interface for 3 to 6 weeks until epithelial differentiation was observed, as indicated by greater than 70% ciliated epithelium under light microscopy as previously described (30). These polarized, differentiated HBECs were then apically infected with 150,000 plaque forming units (PFU) of various strains of IAV for 72 hours and subsequently apically inoculated with 1,000 colony forming units (CFU) of *Spn* serotype 19F, strain EF3030, for 6 hours. Preliminary experiments have shown 48–72 hours to be optimal for logarithmic growth of IAV in HBEC cultures (data not show). Infection with IAV consistently led to increased *Spn* abundance on the apical surface of the HBECs, regardless of IAV strain (A/Puerto Rico/8/1934 [PR8], A/Hong Kong/2/1968 H3N2 [H3N2], or A/California/07/2009 pandemic H1N1 [pH1N1]) (Figure 1A). Notably, the viral strains had different replication kinetics (Supplemental Figure 1A; supplemental material available online with this article; https://doi.org/10.1172/jci.insight.170022DS1) and viral replication was positively correlated with an increase in *Spn* recovered from the airway surface (Figure 1B). This suggested that increased IAV replication facilitates increased *Spn* burden in the ASL. Since the pH1N1 strain of IAV led to the greatest increase in *Spn* burden, we used it as our prototype in subsequent studies hereafter. Additionally, 100,000 PFU of the pH1N1 strain were used in subsequent experiments to account for the higher replication kinetics displayed by this strain of IAV. At this infectious dose, the cytotoxicity of IAV on the airway epithelium was determined to be 15% (Supplemental Figure 1B).

To confirm that active replication of IAV was required to increase *Spn* in the ASL, IAV-infected HBECs were treated with IAV-specific antivirals before inoculation with *Spn*. We took advantage of the distinct mechanisms of action of FDA-approved IAV antivirals to examine the effect of blocking distinct stages of viral replication. HBECs were treated with oseltamivir, which primarily functions by inhibiting late viral egress through inhibition of the neuraminidase protein (31), and baloxavir marboxil, an RNA-dependent RNA polymerase inhibitor that prevents viral replication early during the viral life cycle (32). In contrast to IAV without treatment, which again led to an increase in *Spn* recovered from the airway surface, treatment of HBECs with oseltamivir or baloxavir marboxil 1 hour after IAV infection reduced the burden of *Spn* to the levels seen in HBECs not infected with IAV (Figure 1C). To validate this result, we confirmed that the antiviral treatments effectively prevented IAV replication and could not detect replicating virus after 72 hours of infection in control samples (Supplemental Figure 1C). Next, the timing of the neuraminidase inhibitor oseltamivir was investigated. It is noteworthy that IAV neuraminidase activity has been linked to enhanced adhesion of *Spn* to host cells (14). Additionally, sialic acid released from host cells due to neuraminidase activity serves as a nutrient source for *Spn* (13). Treatment with oseltamivir early in IAV infection again prevented an increase in *Spn*, while treatment immediately before the introduction of *Spn* did not prevent an increase in the bacterial burden (Figure 1D). The inability of IAV neuraminidase inhibition at a later time point to prevent an increase in *Spn* in the ASL suggests that neuraminidase enzymatic activity at the time of *Spn* infection alone is not enough to increase the bacterial burden in the airway epithelium. Collectively, the effect of treatment with antivirals on *Spn* in the airway indicates that permissiveness for viral replication drives the increase in *Spn* after IAV.

### IAV increases susceptibility to bacterial infection throughout the airway.

**Figure 2:**
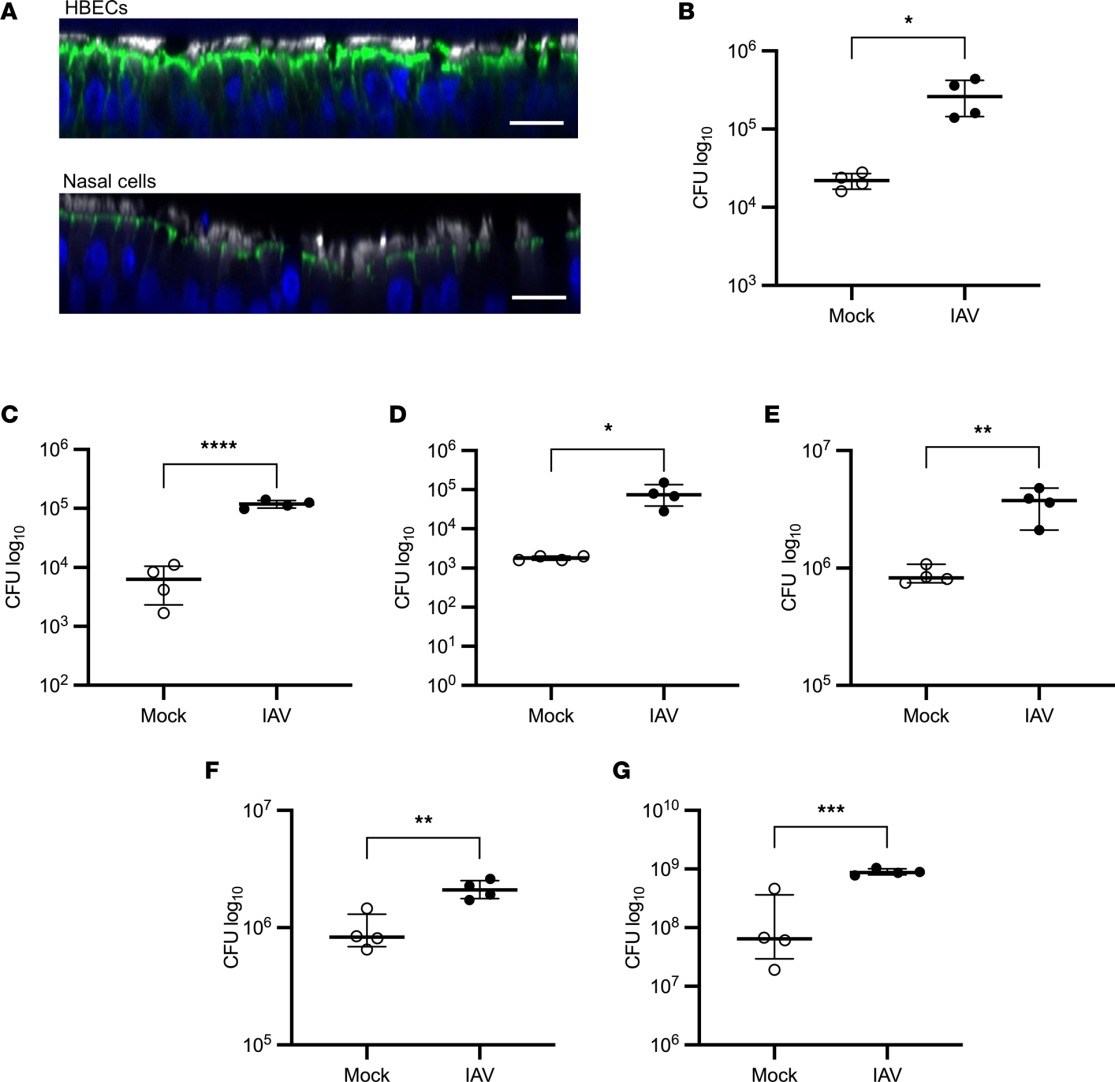
IAV increases the bacterial burden of the ASL in various regions of the airway. (**A**) Uninfected HBECs and primary differentiated human nasal cells were fixed in 4% paraformaldehyde, stained for cilia (white), F-actin (green), and DNA (blue), and imaged at ×40 magnification on a Nikon A1R-HD25 confocal microscope. Scale bars: 20 μm. Images are representative of 3 replicates. (**B**) Primary differentiated human nasal cells, (**C**) primary differentiated small airway epithelial cells, (**D**) HBECs isolated from 15-month-old lungs, (**E**) HBECs isolated from the lungs of a smoker, or (**F**) primary differentiated ferret bronchial epithelial cells were infected with 100,000 PFU of IAV for 72 hours, followed by infection with 1,000 CFU of *Spn*. *Spn* was quantified by vertical plating of apical washed collected after 6 hours of *Spn* infection (*n* = 4). (**G**) HBECs were infected with 100,000 PFU of IAV for 72 hours, followed by infection with 1,000 CFU of *Staphylococcus aureus*. *S*. *aureus* was quantified by vertical plating of apical washed collected after 6 hours of bacterial infection (*n* = 4). Panels **B**–**G** are each representative of 2 independent experiments. All panels were analyzed by unpaired, 2-tailed *t* test. *P < 0.05, **P < 0.01, ***P < 0.001, ****P < 0.0001. Brackets indicate median and interquartile range. Open circles indicate mock IAV infection; closed circles indicate IAV infection.

Different regions of the respiratory tract exhibit many anatomical and cellular similarities but also distinct differences. IAV-mediated susceptibility to *Spn* in anatomically distinct airway epithelial cell types was investigated by isolation of airway epithelial cells from nasal turbinates of human volunteers, tracheobronchial explants, and small airway human explants from which differentiated air-liquid interface cultures were grown. In organotypic air-liquid interface cultures, the nasal airway epithelial cells differentiate to develop pseudostratified columnar morphology, establish an air-liquid barrier, and develop a polarized, ciliated epithelium upon differentiation, similar to tracheobronchial HBECs (Figure 2A). Consistent with previous studies in tracheobronchial-derived airway epithelium, infection with IAV led to an increase in *Spn* in primary differentiated human nasal cells (Figure 2B). The presence of *Spn* did not alter IAV replication (Supplemental Figure 2A). Additionally, when performed on small airway epithelial cells isolated and propagated similarly to HBECs, IAV again increased the burden of *Spn* in the airway epithelium (Figure 2C), further demonstrating a consistent increase in *Spn* at distinct anatomical regions throughout the airway after IAV infection.

IAV exhibits distinct clinical outcomes in different age groups, and this is particularly evident in pediatric populations (33). For this reason, the effect of IAV infection on *Spn* in HBECs isolated from the lungs of a 15-month-old donor was explored. Consistent with previous studies in adult lung–derived HBECs, IAV infection led to a significant increase in *Spn* burden, although *Spn* did not alter IAV replication (Figure 2D and Supplemental Figure 2B). Notably, the HBECs isolated from the lungs of a 15-month-old were less permissive to *Spn* than the HBECs isolated from the lungs of adult donors. This may be due to an increase in airway epithelial barrier function in HBECs isolated from younger donors (34). Additionally, IAV infection in HBECs isolated from the lungs of a smoker led to an increase in *Spn*, showing that an IAV-induced increase in the bacterial burden of the airway is not limited to healthy HBECs (Figure 2E). Finally, in primary differentiated ferret bronchial epithelial cells, IAV infection led to an increase in *Spn* in the ASL (Figure 2F), providing further evidence that IAV consistently enhances *Spn* growth in the airway in other mammalian airways permissive to IAV.

To investigate the effect of IAV infection on the ability of other bacteria to cause secondary infections, the effect of IAV infection on *Staphylococcus aureus* was evaluated. Consistent with previous experiments using *Spn*, IAV infection led to an increase in *Staphylococcus aureus* in the ASL, although the presence of *S*. *aureus* did not alter IAV replication (Figure 2G and Supplemental figure 2C). The increase in *S*. *aureus* after IAV indicates that IAV lowers host defense against bacterial pathogens in the airway and broadly increases susceptibility to secondary bacterial infections.

### IAV profoundly alters the airway epithelium.

**Figure 3:**
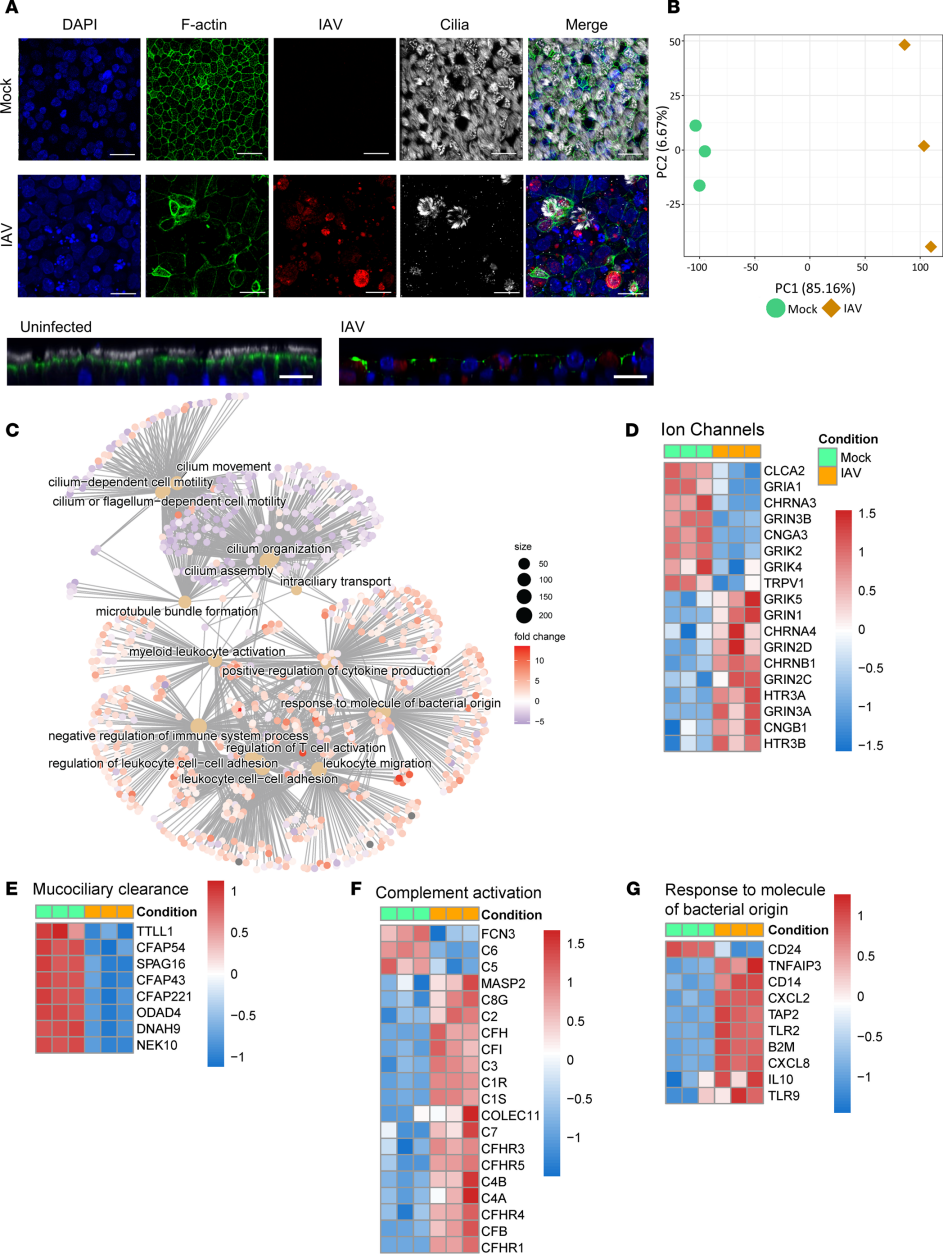
IAV profoundly alters the airway epithelium. HBECs were infected with 100,000 PFU of IAV for 72 hours. (**A**) HBECs were stained for cilia (white), F-actin (green), or IAV (red) and mounted in DAPI Fluoromount (blue). Images were taken at ×40 magnification on a Nikon A1R-HD25. Scale bar: 20 μm. Image is representative of 3 replicates. (**B**) Principal component analysis (PCA) of the transcriptome of HBECs with and without IAV. (**C**) Cnetplot of enriched gene ontology (GO) terms after infection with IAV, with the size of each dot representing the number of differentially expressed (DE) genes in the category and the color indicating log_2_(fold change). The cnetplot was generated using the R package ClusterProfiler v4.0 (**D**–**F**) Heatmap of ion channel (GO: 0015276), mucociliary clearance (GO: 0120197), complement activation (GO: 0006956), and response to a molecule of bacterial origin (GO: 0002237) gene expression in HBECs with and without IAV infection. Transcriptomic analysis was conducted on 3 replicates. Green indicates uninfected and orange indicates IAV infected. DE genes were calculated with a false discovery rate of ≤0.05 and an absolute log_2_(fold change) of ≥1. Plots in **D**–**G** are composed of DE genes from the respective category.

To validate the effect of IAV on the morphologic features of the airway in our model, HBECs were again infected with 100,000 PFU of IAV for 72 hours, followed by infection with 1,000 CFU of *Spn* for 6 hours, and fluorescence microscopy was used to evaluate the effect of IAV on the airway epithelium and *Spn*. IAV caused substantial changes to the airway epithelium, including a loss of cilia (Figure 3A and Supplemental Figure 3), consistent with previous reports (35).

Having rigorously established our HBEC model, the effect of IAV on the transcriptional profile of the airway was investigated to better understand how IAV primes the airway for secondary bacterial infection. Transcriptomics analysis by bulk RNA-seq was performed on 3 technical replicates of HBECs infected with 100,000 PFU of IAV for 72 hours. Principal component analysis revealed a pronounced shift in the transcriptome of the airway epithelium after IAV (Figure 3B). Over 12,000 genes were shown to be differentially regulated between IAV-infected and -uninfected HBECs. The presence or absence of *Spn* had no significant effects on the transcriptomic analysis of either IAV-infected or -uninfected HBECs, mostly likely due to the short duration of bacterial exposure, the low infectious dose used, and the extracellular replication of *Spn* (data not shown). Multiple biological pathways and processes were altered, and evaluation of gene ontology (GO) categories critical to host defense revealed a large number of processes that were diminished (Figure 3C). Consistent with previous findings demonstrating IAV-induced ion channel dysregulation (30), there were alterations in the expression of many ion channels after IAV infection (Figure 3D), although CFTR was not identified. This is consistent with our previous studies implicating posttranslational regulation of CFTR during IAV infection (30). The expression of genes responsible for the maintenance of mucociliary clearance were markedly and uniformly decreased, which is consistent with our fluorescence microscopy findings (Figure 3E) of loss of ciliary axonemes after IAV infection, as previously noted (35). Interestingly, an increase in complement activation after IAV (Figure 3F) was observed by induction of complement cascade transcripts. Complement serves as an essential component of the immune response in the airway epithelium (36), so this alteration may substantially alter the host response bacterial infection. Finally, there was an increase in the expression of many genes in the GO category “response to molecules of bacterial origin” after IAV infection (Figure 3G). The “response molecules of bacterial origin” GO category encompasses broad molecular functions that are altered by the presence of bacteria and bacterially sourced molecules. Changes in this category indicate that IAV-induced changes may alter recognition of bacterial products by IAV-infected HBECs. These findings demonstrate that IAV infection substantially alters the transcriptional profile of the airway epithelium, with many changes leading to diminished epithelial bacterial defense or heightened proinflammatory mechanisms in the airways.

### IAV increases Spn in the airway by inducing CFTR dysfunction.

**Figure 4:**
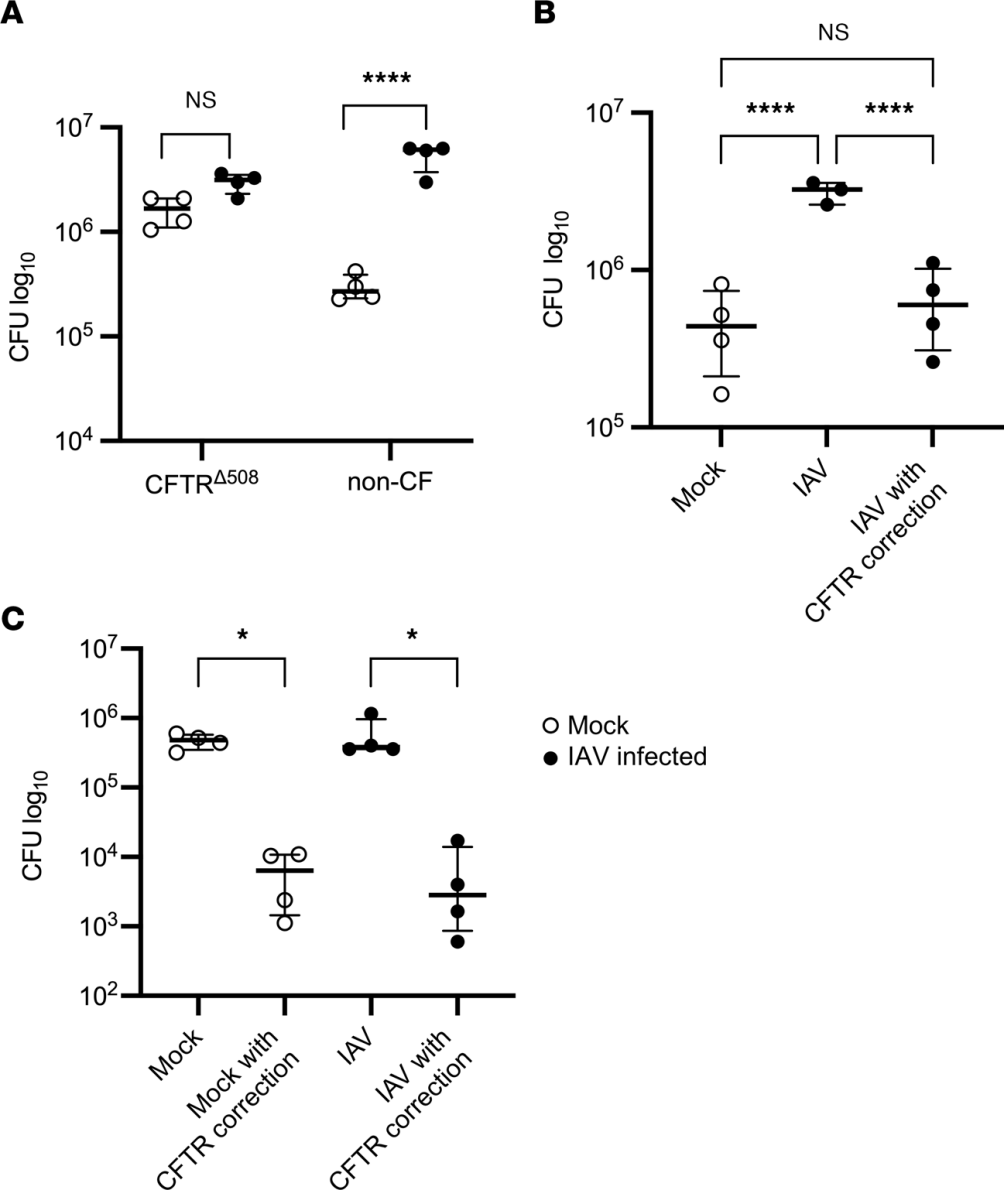
IAV increases *Spn* in the airway by inducing CFTR dysfunction. (**A**) Non-CF and CFTR^Δ508^-HBECs were infected with 100,000 PFU of IAV for 72 hours, followed by infection with 1,000 CFU of *Spn*. *Spn* was quantified by vertical plating of apical washes after 6 hours of *Spn* infection (*n* = 4). (**B**) Non-CF HBECS were infected with IAV and basally treated with 10 μM lumacaftor and tezacaftor at the time of IAV infection, and then basally treated with 10 μM ivacaftor overnight before *Spn* infection. After 72 hours of IAV infection, HBECs were infected with *Spn* for 6 hours. *Spn* was quantified by vertical plating of apical washes (*n* = 3–4). (**C**) CFTR^Δ508^-HBECs were infected with 100,000 PFU of IAV and basally treated with 10 μM lumacaftor and tezacaftor at the time of IAV infection, and then basally treated with 10 μM ivacaftor overnight before *Spn* infection. After 72 hours of IAV infection, HBECs were infected with *Spn* for 6 hours. *Spn* was quantified by vertical plating of apical washes (*n* = 4). Panel **A** is representative of 2 independent experiments, panel **B** is representative of 3 independent experiments, and the experiment in panel **C** was conducted once due to limited availability of CFTR^Δ508^-HBECs. All panels were analyzed by 1-way ANOVA with Bonferroni’s (**A**) or Tukey’s (**B** and **C**) multiple-comparison test. *P < 0.05; ****P < 0.0001. Brackets indicate median and interquartile range. Open circles indicate mock IAV infection; closed circles indicate IAV infection.

Previous research from our laboratory demonstrated that IAV infection causes significant ion channel dysfunction in the airway epithelia, and that this dysfunction alters the rheologic properties of the ASL in intact airways and in vivo (30). In particular, a loss of CFTR function was found to substantially alter the ASL, and correction of CFTR function restored the rheologic properties of the ASL (30). Chronic bacterial infections are a hallmark of ion channelopathies that cause lung disease, such as CF, thus the impact of CFTR dysfunction in IAV-infected HBECs on the development of bacterial infections was investigated. Although there are many different mutations of the CFTR gene that cause CF and their prevalence varies worldwide (37), deletion of phenylalanine 508 (CFTR^Δ508^) represents the most common mutation and leads to misfolding of CFTR and failure of the protein to reach the apical membrane of polarized airway epithelial cells (38). For this reason, HBECs from CF individuals with the CFTR^Δ508^ mutation (designated CFTR^Δ508^-HBECs) were obtained from the lungs of patients with CF undergoing lung transplantation and cultured consistent with our organotypic cultures from non-CF donors. CFTR^Δ508^-HBECs developed similar differentiation characteristics, although they produced more mucus and required more frequent culture maintenance. CFTR^Δ508^-HBECs, when exposed to *Spn* alone, exhibited greater bacterial abundance after 6 hours as compared with non-CF HBECs, consistent with the notion that bacterial susceptibility is increased in CFTR deficiency (Figure 4A). When the effect of IAV on *Spn* was assessed in CFTR^Δ508^-HBECs, IAV did not increase *Spn* in the ASL (Figure 4A), raising the possibility that IAV is not able to increase the burden of *Spn* in HBECs without functional CFTR. Alternatively, the burden of *Spn* in CFTR^Δ508^-HBECs without IAV infection is higher than the baseline in non-CF HBECs and may not be able to be further increased by IAV. These findings indicate that CFTR dysfunction can lead to increased susceptibility to *Spn* growth in human organotypic cultures.

To investigate the role of IAV-induced CFTR dysfunction in susceptibility to *Spn* after IAV, *Spn* was quantified in the ASL after IAV infection and treatment with highly effective modulator therapy (HEMT) consisting of a triple cocktail of CFTR correctors (lumacaftor, tezacaftor) and potentiators (ivacaftor). HEMT treatment of HBECs from non-CF donors did not alter IAV replication, and HEMT treatment in non–IAV-infected conditions did not alter *Spn* (Supplemental Figure 4, A and B). In non-CF HBECs, IAV infection led to a substantial increase in *Spn* in the ASL, as described above. CFTR correction after IAV with HEMT in non-CF HBECs led to a reduction in *Spn* in the ASL and restored it to the levels seen in non–IAV-infected conditions (Figure 4B), indicating that correction of CFTR function after IAV restores host defense against *Spn* in the ASL. Finally, in CFTR^Δ508^-HBECs, treatment with HEMT reduced the burden of *Spn* in the airway both with and without IAV infection (Figure 4C), likely due to HEMT fully restoring the function of CFTR in both uninfected and IAV-infected CFTR^Δ508^-HBECs. This demonstrates a central role for CFTR dysfunction in the increased susceptibility to bacteria following IAV infection.

### IAV acidifies the ASL via CFTR dysfunction.

**Figure 5:**
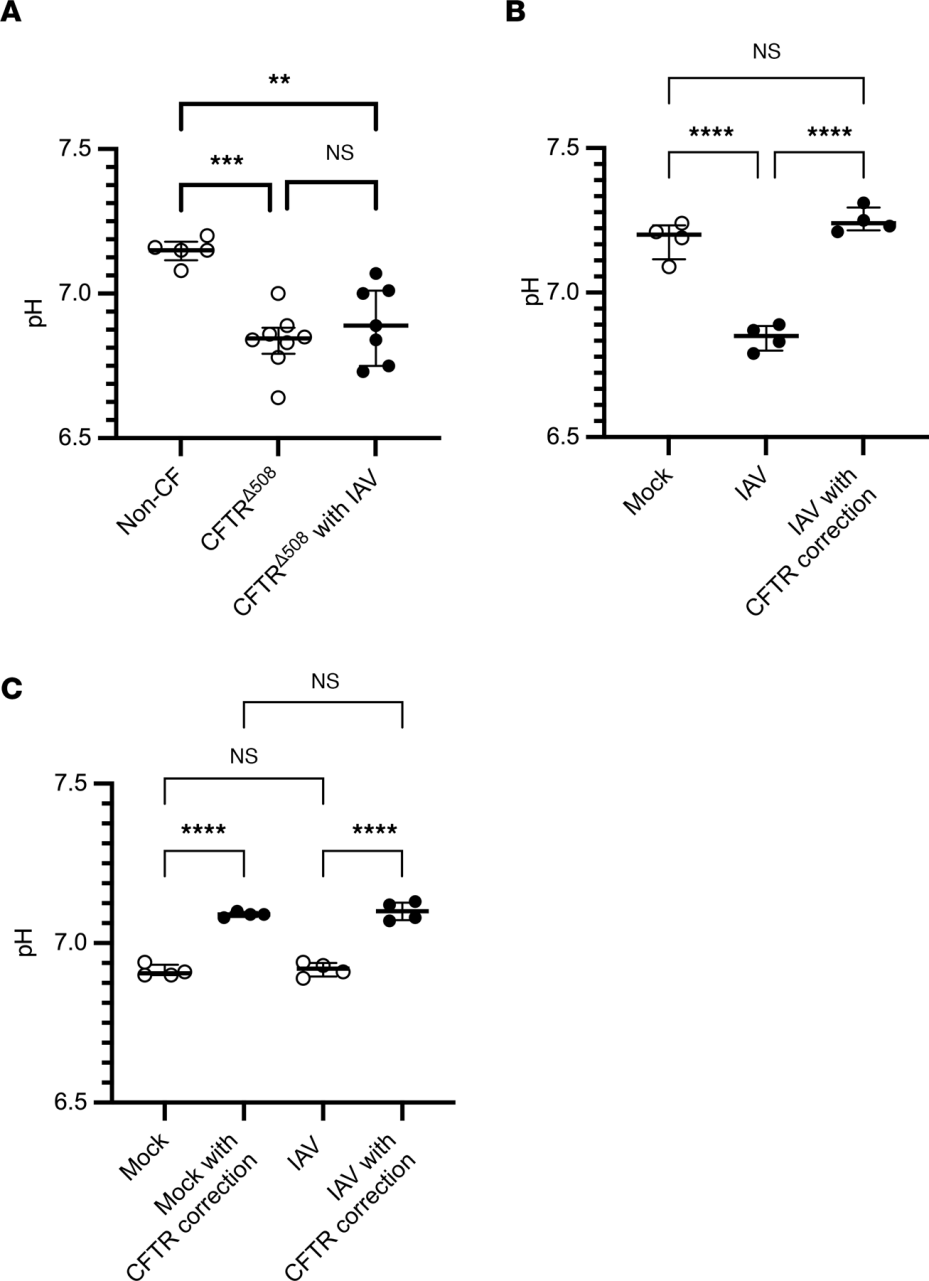
IAV acidifies the ASL by inducing CFTR dysfunction. (**A**) pH measurements were taken of uninfected non-CF and CFTR^Δ508^-HBECs. CFTR^Δ508^-HBECs were infected with 100,000 PFU of IAV for 72 hours before pH measurements were taken (*n* = 5–8). (**B**) Non-CF HBECs were infected with 100,000 PFU of IAV for 72 hours and basally treated with 10 μM lumacaftor and tezacaftor at the time of IAV infection, followed by basal treatment with 10 μM ivacaftor overnight before pH measurements were taken (*n* = 4). (**C**) CFTR^Δ508^-HBECs were infected with 100,000 PFU of IAV for 72 hours and basally treated with 10 μM lumacaftor and tezacaftor at the time of IAV infection, followed by basal treatment with 10 μM ivacaftor overnight before pH measurements were taken (*n* = 4). Panel **B** is representative of 3 independent experiments; experiments in panels **A** and **C** were conducted once due to the limited availability of CFTR^Δ508^-HBECs. All panels were analyzed by 1-way ANOVA with Tukey’s multiple-comparison test. **P < 0.01; ***P < 0.001; ****P < 0.0001. Brackets indicate median and interquartile range. Open circles indicate mock IAV infection; closed circles indicate IAV infection.

CFTR, while primarily known to be a chloride channel, is also known to mediate the transport of bicarbonate, and CFTR dysfunction in CF has been shown to cause acidification of the ASL through regulation of bicarbonate transport (26, 28). To explore the effect of CFTR dysfunction on the pH of the ASL in organotypic HBECs, the pH of the ASL of uninfected CFTR^Δ508^-HBECs and non-CF HBECs was measured using a microelectrode probe. Consistent with previously published research (28), ASL acidification was observed in CFTR^Δ508^-HBECs when compared with non-CF HBECs (Figure 5A). IAV infection of non-CF HBECs significantly reduced the pH of the ASL, lowering it to levels that are consistent with the pH of the ASL in CFTR^Δ508-^HBECs (Figure 5B). HEMT treatment of IAV-infected HBECs raised the pH of the ASL and restored it to the levels seen in noninfected conditions (Figure 5B). Interestingly in CFTR^Δ508^-HBECs, IAV did not lower the pH of the ASL (Figure 5A), raising the possibility that IAV is not able to further acidify the ASL in HBECs with a preexisting CFTR dysfunction. This was further demonstrated by a decrease in the acidity of the ASL after HEMT in CFTR^Δ508^-HBECs in both uninfected and IAV-infected conditions (Figure 5C). This may be due to full restoration CFTR function after HEMT in CFTR-deficient cells, even after the added dysfunction induced by IAV. Finally, transient inhibition of CFTR function by treatment with the CFTR inhibitor 5-[(4-carboxyphenyl)methylene]-2-thioxo-3-[(3-trifluoromethyl)phenyl-4-thiazolidinone (CFTRinh172) led to a moderate acidification of the ASL (Supplemental Figure 5A). Collectively, the effect of CFTR modulation on the pH of the ASL suggests that IAV infection leads to ASL acidification by inducing short-term CFTR dysfunction in airway epithelium.

### Acidification of the ASL caused by IAV increases susceptibility to Spn.

**Figure 6:**
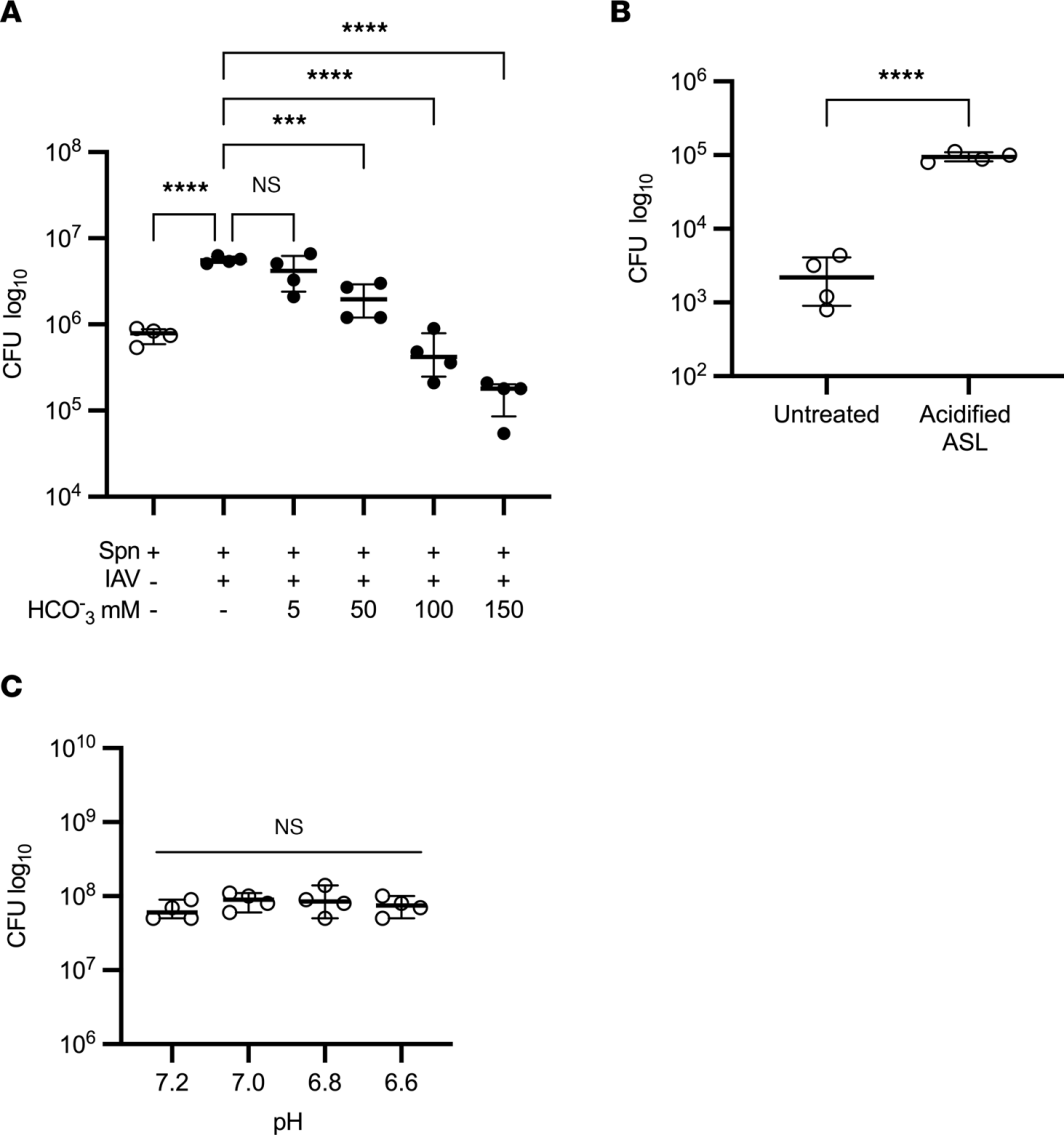
Acidification of the ASL caused by IAV increases susceptibility to *Spn*. (**A**) HBECs were infected with 100,000 PFU of IAV for 72 hours before infection with 1,000 CFU of *Spn* in either physiological saline or isotonic solutions with increasing concentrations of sodium bicarbonate. *Spn* was quantified by vertical plating of apical washes after 6 hours of *Spn* infection (*n* = 4). (**B**) HBECs were infected with 1,000 CFU of *Spn* for 6 hours in either physiological saline or an isotonic buffer at a pH of 6.8. *Spn* was quantified by vertical plating of apical washes (*n* = 4). (**C**) 1,000 CFU of *Spn* was grown overnight in 500 μL of PneumaCult-ALI media at 37°C with 5% CO_2_ and no shaking. The pH of the media was adjusted using 12 M HCl and verified with a pH probe 30 minutes after HCl addition. *Spn* was quantified by vertical plating after overnight growth (*n* = 4). Each panel is representative of 2 independent experiments. Panels **A** and **C** were analyzed by 1-way ANOVA with Dunnett’s (**A**) or Tukey’s (**C**) multiple-comparison test; panel **B** was analyzed by unpaired, 2-tailed *t* test. ***P < 0.001; ****P < 0.0001. Brackets indicate median and interquartile range. Open circles indicate mock IAV infection; closed circles indicate IAV infection.

Acidification of the ASL in CF is known to increase susceptibility to bacterial pathogens in the airway (39, 40). To evaluate the role of ASL acidification in the increase in susceptibility to *Spn* after IAV infection, the apical surface of IAV-infected HBECs was inoculated with *Spn* in physiological saline with an increasing concentration of sodium bicarbonate, a mild base that raises the pH in a dose-dependent manner (data not shown). An increase in sodium bicarbonate concentration led to a corresponding decrease in *Spn*, demonstrating the role of ASL pH in the susceptibility of the airway epithelium to *Spn* (Figure 6A). To evaluate the effect of ASL acidification on *Spn* outside of the context IAV infection, the apical surface of HBECs were inoculated with *Spn* in either physiological saline or an isotonic buffer at a pH of 6.8. Treatment with the mildly acidic buffer led to a significant increase in *Spn* in the ASL (Figure 6B), demonstrating that induced acidification alone increases susceptibility to *Spn* in the ASL. To evaluate the effect of moderate acidification on *Spn* growth outside of the airway, *Spn* was grown in a cell-free environment across a pH range. Changes in pH did not affect *Spn* growth (Figure 6C), suggesting that an increase in *Spn* in the acidified ASL is due to a pH-dependent change in ASL rather than a response of *Spn* to the acidified environment. The effect of pH on *Spn* in the ASL highlights the critical role of IAV-induced changes in the microenvironment of the airway epithelium in increased susceptibility to *Spn* after IAV.

### IAV alters the proteome of the ASL.

**Figure 7:**
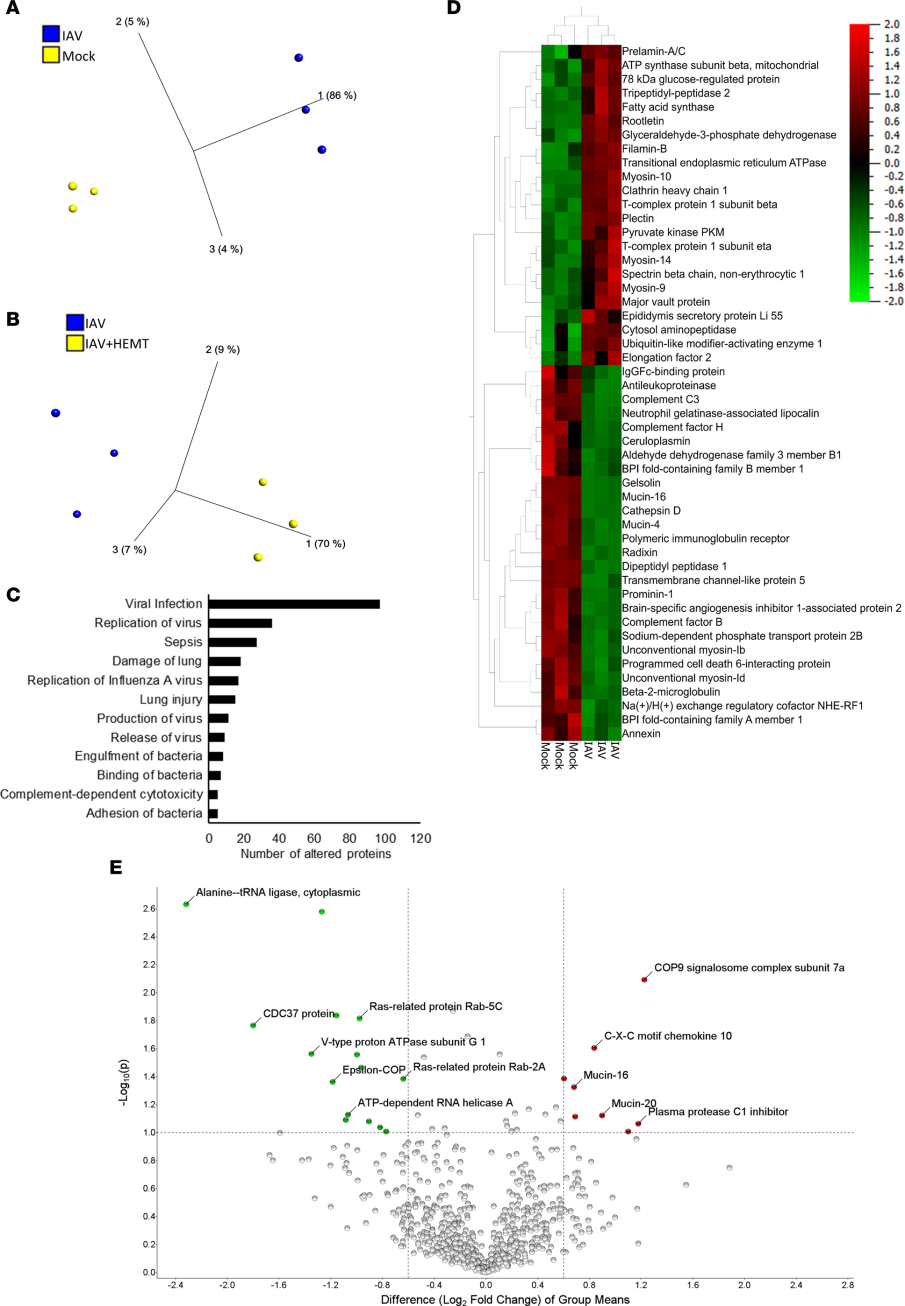
IAV alters the proteome of the ASL. HBECs (*n* = 3) were infected with 100,000 PFU of IAV for 72 hours before apical washes were collected for proteomic analysis. (**A** and **D**) Analysis of the proteome of the ASL of HBECs with and without IAV infection. The following criteria were all met for every reported protein: absolute fold change > 2, significance analysis of microarray > 0.8, and *P* < 0.05. (**B** and **E**) Analysis of the proteome of the ASL from HBECs after IAV infection and with or without basal treatment with 10 μM lumacaftor and tezacaftor at the time of IAV infection followed by basal treatment with 10 μM ivacaftor overnight before ASL collection. The following criteria were met for every reported protein: fold change > 1.5, significance analysis of microarray > 0.6, and *P* < 0.1. (**C**) Bar chart showing the number of proteins in each category altered in the ASL of HBECs after IAV infection. All categories reached statistical significance. Data analysis was done using Qiagen IPA software following the standard statistical analysis protocol.

Given our observations on the negative effects IAV and IAV-induced CFTR dysfunction, we sought to expand our understanding of the effect of IAV infection and subsequent CFTR dysfunction on the proteome of the host airway ASL. HBECs were infected with 100,000 PFU of IAV for 72 hours and apical washes within each biological replicate were pooled for proteomic analysis. This was performed on 3 biological replicates to allow for richer data analysis. Five hundred twenty-one total proteins were identified in every group and biological replicate. Principal component analysis indicated marked differences in the proteomes of the ASL from IAV-infected and noninfected HBECs (Figure 7A). Using stringent criteria (absolute fold change > 2, significance analysis of microarray > 0.8, and *P* < 0.05), IAV significantly increased 82 proteins and decreased 59 proteins in the ASL, further demonstrating the impact of IAV on the microenvironment of the airway epithelium. Correction of CFTR function with HEMT after IAV led to a moderate alteration in protein abundance of the ASL, indicating that CFTR function plays a modest role in the regulation of the proteome of the ASL (Figure 7B). Additionally, substantial changes in various GO terms related to IAV infection, bacterial infection, and lung injury (Figure 7C and Supplemental Table 1) were identified after IAV, demonstrating systemic IAV-induced alterations in the ASL that affect host defense. When evaluating the most substantially altered proteins after IAV, there was a decrease in many proteins that are involved in host defense, including complement proteins, cathepsins, and the serine protease inhibitor antileukoproteinase (Figure 7D). However, CFTR correction did not restore these reduced host defense proteins in the ASL (Figure 7E). This indicates that restoring CFTR function may reduce the bacterial burden of the ASL through functional correction of host defense proteins in the ASL, rather than restoration of the abundance of host defense proteins. Prior research indicates that ASL acidification alters both the function of host defense proteins (29, 40, 41) and the viscosity of mucus in the airway (42, 43). An increase in mucus viscosity may contribute to an increase in *Spn* in the airway epithelium. The effect of IAV on the proteome of the ASL further demonstrates that IAV causes substantial changes in the microenvironment of the airway epithelium that likely have important roles in airway pathophysiology.

## Discussion

While various mechanisms that contribute to an increase in the bacterial burden of the airway after IAV have been studied in animal models, they have not been evaluated in the human airway epithelium. Secondary bacterial infections are a common complication of respiratory viral infections, and lead to significant increases in the morbidity and mortality of the initial respiratory viral infections (1). Here, we show that the airway epithelium plays a critical role in susceptibility to bacterial infection after IAV infection. Importantly, we use a primary differentiated airway epithelial cell culture model to study the airway epithelium in an isolated and biologically relevant manner. This allows us to identify mechanistic changes in the airway epithelium caused by IAV infection that contribute to an increase in *Spn*. While the use of HBECs allows for the study of the airway epithelium in a clinically relevant manner, this model system does present some limitations. The complex nature of the culture system and sample collection limits the number of replicates that can be included in each experiment. There is also a limitation in the number of biological replicates available, although the work in this paper was done with HBECs from 7 different donors. Additionally, our model does not have an adaptive immune system, so we are not able to consider the effect of the immune response in our findings. We find that IAV infection disrupts host defense in the airway by altering the proteome of the ASL and reducing CFTR function, which in turn causes acidification of ASL and reduced bacterial host defense. Induction of CFTR dysfunction in the absence of IAV leads to similar findings, and correction of CFTR function leads to correction of the pH of the ASL and reduced susceptibility to bacterial infections, supporting the hypothesis that CFTR is a central mediator of secondary bacterial infection. These findings are the initial report that CFTR and the airway epithelium intrinsically regulate bacterial susceptibility following influenza infection and puts forth a mechanism for secondary bacterial infections following influenza.

Few studies have addressed the role of the airway epithelium in IAV-mediated pneumococcal infections. IAV increases inflammatory chemokines in the airway and this induced inflammatory state impairs the innate immune response to *Spn*, allowing for an increase in the bacterial burden of the airway (8). IAV also impairs the immune response to *Spn* by inducing a type 1 interferon response and subsequently suppressing the neutrophil clearance of *Spn* (9). Finally, IAV attenuates the immune response to bacterial pathogens by reducing bacterially induced IL-1β signaling and Th-17–mediated immunity (44, 45). However, we observed a consistent increase in *Spn* after IAV infection in our HBEC organotypic model lacking functional innate or adaptive immune cell populations.

Beyond the context of an adaptive immune response, other mechanisms in the airway may contribute to an increase in *Spn*. Synergistic interactions between IAV and *Spn* neuraminidases have been shown to contribute to increased pathogenesis during coinfection (13–15). However, we observed an increase in *Spn* after IAV even after treatment with an IAV neuraminidase inhibitor late in the course of infection, suggesting neuraminidase activity is not the mechanism in our organotypic model. Furthermore, the susceptibility to secondary bacterial infection was found with *S*. *aureus* as well as *Spn,* indicating that the increase in the bacterial burden of the airway is not *Spn* specific. When treated with different FDA-approved antivirals early in infection, IAV did not to increase susceptibility to bacterial infection, indicating that replication is required. Finally, IAV has been shown to reduce mucociliary clearance in the airway, reducing bacterial clearance (20). In our model, although there is mucociliary action, there is no mucociliary clearance because the HBECs are grown and infected in a closed in vitro system, suggesting that other IAV-induced changes in the airway epithelium may contribute to an increase in the burden of *Spn* in the ASL.

Utilizing both fluorescence microscopy and transcriptomics analysis, we found that IAV causes significant morphological and phenotypical damage in the airway epithelium consistent with increased susceptibility to secondary bacterial infection. Among these is a consistent loss of cilia and reduced expression of genes responsible for cilia structure and function. Additionally, many of the transcriptional changes after IAV involve host genes that play key roles in defense against bacterial pathogens. These findings suggest that IAV-mediated transcriptional changes may lead to a reduction in host defense mechanisms against bacterial pathogens. Consistent with prior research from our lab (30), there was a substantial change in the expression of ion channels after IAV.

Based on our initial findings, we further investigated the role of a reduction in function of the CFTR ion channel, which plays a key role in the maintenance of homeostasis in the airway epithelium. Prior research has shown that the matrix protein 2 encoded by IAV reduces CFTR abundance and function in IAV-infected cells by targeting CFTR for ubiquitination and subsequent lysosomal degradation (46, 47). Recurrent and chronic high bacterial loads in the airway is one of the hallmarks of CF disease caused by genetically driven CFTR dysfunction (23). For this reason, we hypothesized that short-term CFTR dysfunction during IAV infection could increase *Spn* susceptibility. Consistent with this notion, restoring CFTR function after IAV infection decreased the abundance of *Spn* in the ASL, commensurate with previous findings that therapeutic restoration of CFTR function reduces the bacterial burden in the airway of CF patients (48).

Based on these observations, we sought to define the mechanism of IAV-induced CFTR dysfunction that was allowing for an increase in the bacterial burden of the airway. CF is characterized by a chronic yet ineffective inflammation (49); however, our model lacks functional immune cell populations. Multiple lines of evidence have shown that CFTR dysfunction reduces the depth of the ASL, and this is consistent with previous research from our lab showing that IAV-induced CFTR dysfunction reduces the depth of the ASL (30). Dehydration of the ASL has been shown to cause a worsening of the diseases state of CF and an increase in the bacterial burden of the airway (24, 50). This dehydration also causes a reduction in mucociliary clearance, preventing the removal of invading pathogens (51). However, in our model, the ASL is rehydrated with physiological saline by the application of bacteria to the apical surface; thus, dehydration is not likely a mechanism for our findings. Additionally, our model is a closed system, so while there is mucociliary action, there is no mucociliary clearance.

CFTR has been shown to control ASL pH in HBECs (28), and the acidification of the ASL in CF has been shown to contribute to an increase in the bacterial burden of the airway (27, 29). Against this background, we hypothesized that IAV-induced CFTR dysfunction would acidify the ASL. As hypothesized, IAV infection caused an acidification of the ASL in non-CF HBECs, and restoration of CFTR function after IAV restored the pH of the ASL to the levels of healthy uninfected cells. This is consistent with prior research showing that correction of CFTR function in CFTR^Δ508^-HBECs corrected ASL acidification (52). We then hypothesized that IAV-induced ASL acidification contributes to the increase in bacterial burden after IAV. ASL acidification increased *Spn* in the ASL, and correction of the pH of the ASL after IAV reduced the bacterial burden of the ASL. This is consistent with previous research showing that ASL acidification in CF causes a loss of host defense and increase in the bacterial burden of the ASL (27, 29, 40).

Host defense proteins found in the healthy ASL serve an essential role in the control of bacteria in the lungs because they are broadly effective against bacterial pathogens and are constitutively present at the time of infection (53). For this reason, we investigated the effect of IAV infection and subsequent CFTR dysfunction on the proteomic profile of the ASL. IAV infection caused a significant change in the proteome of the ASL, including many proteins with known host defense properties. Additionally, CFTR correction after IAV again led to a change in the proteome of the ASL but did not restore it to the state seen in uninfected HBECs. This indicates that the changes in the microenvironment of the ASL caused by CFTR dysfunction may cause a reduction in the function of host proteins without significantly altering the abundance of these proteins. Many of the essential host defense proteins in the ASL are pH dependent and may lose function in an acidified airway environment, including lactoferrin, transferrin, β-defensin 3, and LL-37 (41, 54–56). ASL pH is also known to alter mucus viscosity in CF (42, 43), and this altered viscosity may play a role in increased *Spn* after IAV infection.

Finally, the changes observed in the proteome of the ASL did not always align with the differential gene expression seen in the transcriptomics analysis, as is seen in the increase in expression of complement genes and decrease in the abundance of complement proteins in the ASL. This indicates that IAV is altering the microenvironment of the host airway both transcriptionally and through nontranscriptionally dependent mechanisms, likely through regulation of host protein function independently of abundance. Additionally, while our transcriptomic analysis was conducted on the airway epithelium, our proteomic analysis involved only the apically secreted proteins. This may account for additional discrepancies between the data sets, particularly those involving nonsecreted proteins.

Our work is the first to our knowledge to demonstrate that the airway epithelium plays a critical role in the development of secondary bacterial infections following influenza. We find that IAV infection consistently increases the bacterial burden of the human airway epithelium, that this increase is conveyed by various influenza strains, and that this increase is dependent on IAV-induced CFTR dysfunction and subsequent ASL acidification. Transcriptomic and proteomic analysis identifies multiple epithelium-driven host defense mechanisms that are compromised by influenza infection. Future studies of bacterial complications secondary to influenza infection should incorporate epithelial mechanisms as critical initial alterations leading to bacterial susceptibility.

## Methods

### Cell culture.

Madin-Darby canine kidney cells (MDCK, CCL-34, American Type Culture Collection) were maintained in 1× minimum essential media (MEM, Gibco) supplemented with 10% fetal bovine serum (Invitrogen) and 100 μg/mL streptomycin (Antimycotic-Antibiotic, Invitrogen) at 37°C with 5% CO_2_. Cells were lifted for passaging using 0.25% trypsin-EDTA (Gibco) and discarded after 12 passages.

HBECs were isolated from human lung tissue provided by the International Institute for the Advancement of Medicine (IIAM) and collected as previously described (57). Lungs were provided from both male and female donors, and HBECs from both sexes were used throughout the manuscript. Isolated cells were plated onto 100 mM collagen–coated dishes (Advanced Biomatrix) and grown to confluence in BronchiaLife Complete Growth Media (LifeLine Cell Technologies). Cells were passaged using 0.25% trypsin-EDTA (Gibco) onto 6.5 mm permeable supports with a 4 μm pore size (Costar) coated with FNC coating mix (AthenaES). Once the cells reached confluence on the permeable supports, growth media was removed from the apical surface and the cells were differentiated by apical air exposure and basal treatment with PneumaCult-ALI supplemented basal medium (StemCell Technologies). Basal media was replaced every day for 1 week. After 1 week on air, basal media was replaced and the apical surface of the cells were washed with 200 μL PBS (Gibco) to remove excess mucus 3 times per week. Cells were observed for the presence of cilia and mucus production after 2 weeks on air, and experiments were conducted after the observation of these markers of differentiation. The work presented in this paper used cells isolated from 7 different human donor lungs.

### Viral culture.

IAV pH1N1 (obtained from the Center for Disease Control and Prevention, Atlanta), IAV PR/8, and IAV H3N2 (BEI Resources) were propagated by infecting MDCK cells at an MOI of 0.01 to 0.1 for 48 hours at 37°C with 5% CO_2_ in 1× serum-free Opti-MEM (Gibco) supplemented with 100 μg/mL streptomycin (Antimycotic-Antibiotic, Invitrogen) and 3 μg/mL TPCK-treated trypsin (Worthington Biochemical Corporation). Virus was quantified by plaque assay on MDCK cells. For plaque assay analysis, MDCK cells were infected in 1× serum-free MEM supplemented with 100 μg/mL Antimycotic-Antibiotic and 3 μg/mL TPCK-treated trypsin. After 1 hour of infection, an overlay of 0.6% Avicel (FMC Biopolymer) was added. After 48 hours of incubation, the overlay was removed and cells were fixed with 10% neutral buffered formalin (Fisher Chemical) for 1 hour. Formalin was removed and cells were stained with 0.05% neutral red (Sigma-Aldrich).

### IAV infection of differentiated airway cells.

After 18 to 28 days on air and epithelial differentiation as determined under light microscopy, HBECs were apically washed with 200 μL of PBS and given new basal media. They were then apically infected with 100,000–150,000 PFU of IAV suspended in 20 to 100 μL of PBS or physiological saline and incubated at 37°C with 5% CO_2_. This infectious dose and time course were selected because they provide optimal replication kinetics without causing excessive cell death (data not shown). After 48 to 80 hours of IAV infection, apical washes were collected in 400 μL of PBS and frozen at –80°C. IAV was quantified by foci-forming assay of the apical wash. The pH of the ASL was measured using a micro pH electrode (Thermo Fisher Scientific). HBECs used for fluorescence microscopy were fixed in 4% paraformaldehyde (Electron Microscopy Sciences). HBECs used for transcriptomic analysis were preserved in RNAprotect (Qiagen). For drug treatments, HBECs were basally treated with tezacaftor, lumacaftor, and ivacaftor or CFTR inhibitor 172 with time course and concentration indicated in figure legends. The dose of CFTR inhibitor 172 was selected based on previously published research (58). CFTR potentiator and corrector doses were selected based on preliminary data indicating maximal efficacy and minimal cytotoxicity at these doses (data not shown). For foci-forming assay analysis, MDCK cells were inoculated with apical wash diluted in 1× serum-free MEM supplemented 100 μg/mL Antimycotic-Antibiotic and 3 μg/mL TPCK-treated trypsin. After 1 hour of infection, an overlay of 0.6% Avicel was added. After overnight incubation, the overlay was removed, and cells were fixed with 10% neutral buffered formalin for 1 hour. Formalin was removed and cells were permeabilized with 0.5% H_2_O_2_ (Fisher Bioreagents) in methanol (Fisher Chemical). Cells were then blocked in Blotto (Rockland Antibodies and Assays) washed and stained with a 1:1000 dilution of mouse anti-IAV primary antibody (MAB8251, EMD Millipore) in Blotto for 1 hour. Cells were washed 5 times in PBS and stained with a 1:1000 dilution of goat anti-rabbit–HRP secondary antibody (QAB10257, Quirebio). Cells were washed and staining was developed with ImmPACT DAB substrate kit (Vector Laboratories).

### Bacterial infection of differentiated airway cells.

After 48 to 72 hours of IAV infection, HBECs were apically inoculated with 1,000 CFU of *Spn* or *S*. *aureus* suspended in 20 μL of physiological saline, an isotonic buffer at a pH of 6.8, or a sodium bicarbonate solution and incubated for 6 hours at 37°C with 5% CO_2_. This infectious dose and time course were selected to allow for maximal growth of the bacteria with minimal damage to the HBECs. Sodium levels in the bicarbonate solution were adjusted to maintain an isotonic state. After incubation, apical washes were collected in 400 μL of physiological saline. *Spn* in the apical wash was quantified by vertical plating of the apical wash on sheep’s blood agar plates (Remel) to allow for the observation of the characteristic hemolysis.

### Fluorescence microscopy.

HBECs were fixed overnight in 4% paraformaldehyde (Electron Microscopy Sciences) and washed with PBS. Cells were then permeabilized with 0.1% Triton X-100 (PerkinElmer) in PBS for 10 minutes and blocked with 5% heat-denatured donkey serum (Jackson ImmunoResearch) in PBS for 30 minutes. HBECs were stained with primary antibodies against α-tubulin (rabbit monoclonal antibody 5335S, Cell Signaling Technology), IAV (mouse monoclonal antibody MAB8251, EDM Millipore), and F-actin conjugated with fluorophore 488 (Invitrogen A12379, Alexa Fluor 488 phalloidin) diluted 1:500 in heat-denatured donkey serum for 1 hour and then washed 3 times with PBS. HBECs were then stained with a secondary donkey anti-rabbit antibody conjugated with fluorophore 647 (Abcam, ab150075) and a secondary donkey anti-mouse antibody conjugated with fluorophore 546 (Abcam, ab175472) diluted 1:1000 in heat-denatured donkey serum for 30 minutes and washed 3 times in PBS. After staining, cell layers were mounted in DAPI Fluoromount-G (Southern Biotech) on slides and dried overnight at room temperature. Cell layers were imaged on a Nikon A1R-HD25 confocal microscope system using a Plan Fluor 40× DIC h n2 NA 1.3 we 240 μm objective and processed on the Nis Elements 5.0 imaging software. Cilia quantification was performed using ImageJ (NIH).

### Proteomics analysis.

Our previous experience with proteomics analysis of the ASL from HBECs had been discouraging, as the high levels of albumin in the media had passed through to the apical surface and led to difficulty in assessment of HBEC-derived proteins in the ASL. For this reason, HBECs were moved to 1× serum and albumin-free MEM (Gibco) supplemented with 100 μg/mL Antimycotic-Antibiotic 2 days before IAV infection to minimize albumin in the ASL and allow for proteomics analysis. Cells were given new basal media and infected with 100,000 PFU of IAV for 72 hours at 37°C with 5% CO_2_. After IAV infection, apical washes were collected in 80 μL of PBS. Washes were collected and pooled from 4 wells by using the same volume of PBS to wash each well. This provided a sample with a high protein concentration to allow for proteomics analysis. Washes were frozen at –80°C until proteomic analysis was performed.

Protein was extracted using the M-PER Mammalian Protein Extraction Reagent (Thermo Fisher Scientific) and protein content of the apical washes was quantified by Pierce bicinchoninic acid assay (Thermo Scientific). Proteins were diluted in NuPAGE LDS sample buffer (Invitrogen), reduced with dithiothreitol, and denatured at 70°C for 10 minutes. Proteins were then separated on a Novex NuPage 10% Bis-tris gel (Invitrogen), stained overnight with Novex Colloidal Blue Staining kit (Invitrogen), destained, cut into fractions, and digested with trypsin. Peptide digests were reconstituted in 0.1% formic acid (FA)/ddH_2_O at 0.1 μg/μL. Digests were injected onto a 1260 Infinity nHPLC stack (Agilent Technologies) and separated using a 75 micron ID × 15 cm pulled-tip C-18 column (Jupiter C-18 300 Å, 5 micron, Phenomenex) and run in-line on the Thermo Orbitrap Q Exactive HF-X set to the collision-induced dissociation mode and equipped with the Nanospray Flex ion source (Thermo Fisher Scientific). The nHPLC was configured with binary mobile phases that includes solvent A (0.1% FA in ddH_2_O), and solvent B (0.1% FA in 15% ddH_2_O/85% acetonitrile), programmed as follows: 10 minutes at 5% B (2 μL/ min, load); 90 minutes at 5% to 40% B (linear: 0.5 nL/min, analyze); 5 minutes at 70% B (2 μL/min, wash); and 10 minutes at 0% B (2 μL/min, equilibrate). Following each parent ion scan (300–1200 *m*/*z* at 60k resolution), fragmentation data (MS2) was collected on the top most intense 20 ions at 7.5k resolution. For data-dependent scans, charge-state screening and dynamic exclusion were enabled with a repeat count of 2, repeat duration of 30 seconds, and exclusion duration of 90 seconds. Mass spectrometry data were analyzed using MascotServer (Matrix Science) to generate peptide IDs. Peptide analysis was done using the Scaffold 5 software (Protein Sciences). Analysis was done with filter cutoff values that included a minimal peptide length of more than 5 AA’s, with no MH+1 charge states, with peptide probability CIs of greater than 80%, and with the number of peptides per protein of 2 or greater. The protein probability was set to a CI of greater than 99.0%, and a false discovery rate of less than 1.0. Spectral count abundances were normalized between samples prior to further analysis. GO analysis was conducted using IPA software (Qiagen) and the standard statistical analysis.

### Transcriptomics data collection and analysis.

HBECs preserved in RNAprotect (Qiagen) were pelleted by centrifugation at 10,000 rpm. Cell pellets were incubated in 100 μL lysis buffer (10 μL of mutanolysin, 20 μL of proteinase K, 30 μL of lysozyme, 40 μL of TE buffer) for 10 minutes followed by RNA isolation using the RNeasy mini kit (Qiagen). RNA was analyzed using a bioanalyzer and ribosomal RNA was depleted using the RiboZero rRNA removal kit (Illumina). RNA libraries were constructed using the NEBnext Ultra Directional RNA Library prep kit (New England Biolabs) on approximately 1.0 μg of enriched mRNA. cDNA was purified and library size selection was done using the AMPure SpriSelect beads (Beckman Coulter Genomics). Libraries were assessed using the LabChip GX (PerkinElmer) and the Library Quantification Kit (Roche). RNA-seq was done using the Illumina NovaSeq 6000 platform on 150-nt pair-end runs, with 2 to 3 replicates per condition.

FASTQ files were mapped to the human genome GRCh38 assembly GCA_000001405.15 using HISAT2. Gene expression counts were estimated using hTseq. Differentially expressed (DE) genes were determined using the R package DESeq2 using noninfected controls as the baseline and filtered with a false discovery rate cutoff of 0.05 or lower and an absolute log_2_(fold change) cutoff of 1 or greater. Heatmaps of DE genes were generated based on *z* scores of variance-stabilized transformation counts generated in the DESeq2 R package. DE gene lists were used to determine GO biological processes using R package ClusterProfiler v4.0.

### Statistics.

Comparisons between more than 2 groups were made using 1-way ANOVA with Tukey’s or Dunnett’s test for multiple comparisons. Single comparisons were made using a 2-tailed, unpaired *t* test. A *P* value of less than 0.05 was considered significant. Correlation analysis was done using Pearson’s correlation analysis with a CI of 95%.

### Study approval.

All protocols using human tissue were reviewed by the University of Alabama at Birmingham (UAB) IRB as exempt as deidentified tissue procured through the International Institute for the Advancement of Medicine.

### Data availability.

Data used for transcriptomics analysis are available in the NCBI Gene Expression Omnibus (GEO GSE225108) at https://www.ncbi.nlm.nih.gov/geo/query/acc.cgi?acc=GSE225108

## Author contributions

EYE and KSH designed experiments and wrote and edited the manuscript. EYE, JLT, ADM, MYJ, ESC, and JAM designed and conducted experiments and analyzed data. CJO and HT provided conceptual guidance for experiments and edited the manuscript. ADM, HT, and JAM performed bioinformatics analysis.

## Supplementary Material

Supplemental data

Supporting data values

## Version 1 06/15/2023

In-Press Preview

## Version 2 07/24/2023

Electronic publication

## References

[1] Liu Y (2021). Outcomes of respiratory viral-bacterial co-infection in adult hospitalized patients. EClinicalMedicine.

[2] Collaborators GBDLRI (2018). Estimates of the global, regional, and national morbidity, mortality, and aetiologies of lower respiratory infections in 195 countries, 1990-2016: a systematic analysis for the Global Burden of Disease Study 2016. Lancet Infect Dis.

[3] Klein EY (2016). The frequency of influenza and bacterial coinfection: a systematic review and meta-analysis. Influenza Other Respir Viruses.

[4] Bartley PS (2021). Bacterial coinfection in influenza pneumonia: Rates, pathogens, and outcomes. Infect Control Hosp Epideniol.

[5] Chien YW (2009). Bacterial pathogens and death during the 1918 influenza pandemic. N Engl J Med.

[6] Morens DM (2008). Predominant role of bacterial pneumonia as a cause of death in pandemic influenza: implications for pandemic influenza preparedness. J Infect Dis.

[7] Palacios G (2009). Streptococcus pneumoniae coinfection is correlated with the severity of H1N1 pandemic influenza. PLoS One.

[8] Jochems SP (2018). Inflammation induced by influenza virus impairs human innate immune control of pneumococcus. Nat Immunol.

[9] Shahangian A (2009). Type I IFNs mediate development of postinfluenza bacterial pneumonia in mice. J Clin Invest.

[10] Sun K, Metzger DW (2008). Inhibition of pulmonary antibacterial defense by interferon-gamma during recovery from influenza infection. Nat Med.

[11] Ghoneim HE (2013). Depletion of alveolar macrophages during influenza infection facilitates bacterial superinfections. J Immunol.

[12] Wu Y (2015). Lethal coinfection of influenza virus and Streptococcus pneumoniae lowers antibody response to influenza virus in lung and reduces numbers of germinal center B cells, T follicular helper cells, and plasma cells in mediastinal lymph Node. J Virol.

[13] Siegal SJ (2014). Influenza promotes pneumococcal growth during coinfection by providing host sialylated substrates as a nutrient source. Cell Host & Microbe.

[14] McCullers JA (2003). Role of neuraminidase in lethal synergism between influenza virus andstreptococcus pneumoniae. J Infect Dis.

[15] Wren JT (2017). Pneumococcal Neuraminidase A (NanA) promotes biofilm formation and synergizes with influenza A virus in nasal colonization and middle ear infection. Infect Immun.

[16] Sender V (2020). Capillary leakage provides nutrients and antioxidants for rapid pneumococcal proliferation in influenza-infected lower airways. Proc Natl Acad Sci U S A.

[17] Rowe HM (2019). Direct interactions with influenza promote bacterial adherence during respiratory infections. Nat Microbiol.

[18] Platt MP (2022). A multiomics analysis of direct interkingdom dynamics between influenza A virus and Streptococcus pneumoniae uncovers host-independent changes to bacterial virulence fitness. PLoS Pathog.

[19] Smith AP (2021). Dynamic pneumococcal genetic adaptations support bacterial growth and inflammation during coinfection with influenza. Infect Immun.

[20] Pittet LA (2010). Influenza virus infection decreases tracheal mucociliary velocity and clearance of *Streptococcus pneumoniae*. Am J Respir Cell Mol Biol.

[21] Guo J (2022). Worldwide rates of diagnosis and effective treatment for cystic fibrosis. J Cyst Fibros.

[22] Perez A (2007). CFTR inhibition mimics the cystic fibrosis inflammatory profile. Am J Physiol Lung Cell Mol Physiol.

[23] Zemanick ET (2017). Airway microbiota across age and disease spectrum in cystic fibrosis. Eur Respir J.

[24] Donaldson SH (2006). Mucus clearance and lung function in cystic fibrosis with hypertonic saline. N Engl J Med.

[25] Gharib SA (2009). Mapping the lung proteome in cystic fibrosis. J Proteome Res.

[26] Poulsen JH (1994). Bicarbonate conductance and pH regulatory capability of cystic fibrosis transmembrane conductance regulator. Proc Natl Acad Sci U S A.

[27] Simonin J (2019). Airway surface liquid acidification initiates host defense abnormalities in cystic fibrosis. Sci Rep.

[28] Coakley RD (2003). Abnormal surface liquid pH regulation by cultured cystic fibrosis bronchial epithelium. Proc Natl Acad Sci U S A.

[29] Shah VS (2016). Airway acidification initiates host defense abnormalities in cystic fibrosis mice. Science.

[30] Brand JD (2018). Influenza-mediated reduction of lung epithelial ion channel activity leads to dysregulated pulmonary fluid homeostasis. JCI Insight.

[31] Davies BE (2010). Pharmacokinetics of oseltamivir: an oral antiviral for the treatment and prophylaxis of influenza in diverse populations. J Antimicrob Chemother.

[32] Todd B (2021). The active form of the influenza cap-snatching endonuclease inhibitor baloxavir marboxil is a tight binding inhibitor. J Biol Chem.

[33] Leung CH (2014). Clinical characteristics of children and adults hospitalized for influenza virus infection. J Microbiol Immunol Infect.

[34] De Vries M (2022). The relation between age and airway epithelial barrier function. Respir Res.

[35] Wu NH (2016). The differentiated airway epithelium infected by influenza viruses maintains the barrier function despite a dramatic loss of ciliated cells. Sci Rep.

[36] Kulkarni HS (2018). The complement system in the airway epithelium: An overlooked host defense mechanism and therapeutic target?. J Allergy Clin Immunol.

[37] https://apps.who.int/iris/handle/10665/68702.

[38] Lukacs GL, Verkman AS (2012). CFTR: folding, misfolding and correcting the ΔF508 conformational defect. Trends Mol Med.

[39] Pezzulo AA (2012). Reduced airway surface pH impairs bacterial killing in the porcine cystic fibrosis lung. Nature.

[40] Nakayama K (2002). Acid stimulation reduces bactericidal activity of surface liquid in cultured human airway epithelial cells. Am J Respir Cell Mol Biol.

[41] Abou Alaiwa MH (2014). pH modulates the activity and synergism of the airway surface liquid antimicrobials beta-defensin-3 and LL-37. Proc Natl Acad Sci U S A.

[42] Ferrera L (2021). The application of bicarbonate recovers the chemical-physical properties of airway surface liquid in cystic fibrosis epithelia models. Biology (Basel).

[43] Tang XX (2016). Acidic pH increases airway surface liquid viscosity in cystic fibrosis. J Clin Invest.

[44] Kudva A (2011). Influenza A inhibits Th17-mediated host defense against bacterial pneumonia in mice. J Immunol.

[45] Robinson KM (2013). Influenza A exacerbates Staphylococcus aureus pneumonia by attenuating IL-1β production in mice. J Immunol.

[46] Londino JD (2013). Influenza matrix protein 2 alters CFTR expression and function through its ion channel activity. Am J Physiol Lung Cell Mol Physiol.

[47] Londino JD (2015). Influenza virus M2 targets cystic fibrosis transmembrane conductance regulator for lysosomal degradation during viral infection. FASEB J.

[48] Graeber SY (2021). Effects of lumacaftor-ivacaftor on lung clearance index, magnetic resonance imaging, and airway microbiome in Phe508del homozygous patients with cystic fibrosis. Ann Am Thorac Soc.

[49] Cantin AM (2015). Inflammation in cystic fibrosis lung disease: Pathogenesis and therapy. J Cyst Fibros.

[50] Matsui H (2006). A physical linkage between cystic fibrosis airway surface dehydration and Pseudomonas aeruginosa biofilms. Proc Natl Acad Sci U S A.

[51] Matsui H (1998). Evidence for periciliary liquid layer depletion, not abnormal ion composition, in the pathogenesis of cystic fibrosis airways disease. Cell.

[52] Luckie DB (2014). Chemical rescue of ΔF508-CFTR in C127 epithelial cells reverses aberrant extracellular pH acidification to wild-type alkalization as monitored by microphysiometry. Biochem Biophys Res Commun.

[53] Geitani R (2020). Expression and roles of antimicrobial peptides in innate defense of airway mucosa: potential implication in cystic fibrosis. Front Immunol.

[54] Rastogi N (2016). Structure of iron saturated C-lobe of bovine lactoferrin at pH 6.8 indicates a weakening of iron coordination. Proteins.

[55] Barros CA (2021). Influence of iron binding in the structural stability and cellular internalization of bovine lactoferrin. Heliyon.

[56] Abdizadeh H (2015). Detailed molecular dynamics simulations of human transferrin provide insights into iron release dynamics at serum and endosomal pH. J Biol Inorg Chem.

[57] Fulcher ML (2005). Well-differentiated human airway epithelial cell cultures. Methods Mol Med.

[58] Chang J (2020). Ion transport mechanisms for smoke inhalation-injured airway epithelial barrier. Cell Biol Toxicol.

