# Supplementary material for: Influenza A–induced cystic fibrosis transmembrane conductance regulator dysfunction increases susceptibility to Streptococcus pneumoniae
Source: JCI Insight. 2023 Jul 24;8(14):e170022. doi: 10.1172/jci.insight.170022 (PMC10443798; doi:10.1172/jci.insight.170022)
Supplement: Supplemental data [file jciinsight-8-170022-s038.pdf]

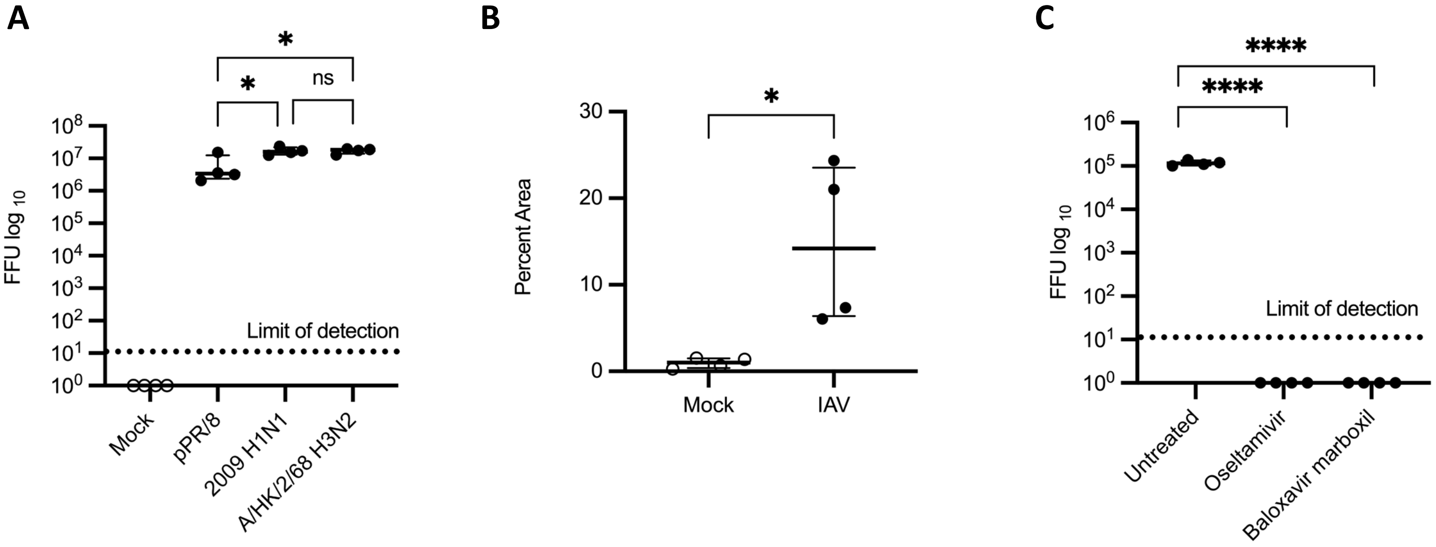

Supplemental Figure 1 (**A**) HBECS were infected with 150,000 PFU IAV for 72 hours, followed by infection with 1,000 CFU *Spn*. IAV was quantified by foci assay of apical washes collected after six hours of *Spn* infection. (**B**) HBECs were infected with 100,000 PFU of IAV (pH1N1) for 72 hours, then stained with trypan blue to quantify cell death. Images were obtained using the Leica DMi1 at 5X magnification and trypan blue staining was quantified using Image J. (**C**) HBECs were infected with 100,000 PFU of IAV and treated basally with 1mM oseltamivir or 100nM baloxavir marboxil for 72 hours, followed by infection with 1,000 CFU *Spn.* IAV was quantified by foci assay of apical washes collected after six hours of *Spn* infection. Panels A and C were analyzed by one-way ANOVA. Panel B was analyzed by unpaired t-test. *, **, ***, **** indicates p of < 0.05, 0.01, 0.001, 0.0001 respectively. Brackets indicate median and interquartile range. Open circles indicate mock IAV infection, closed circles indicate IAV infection.

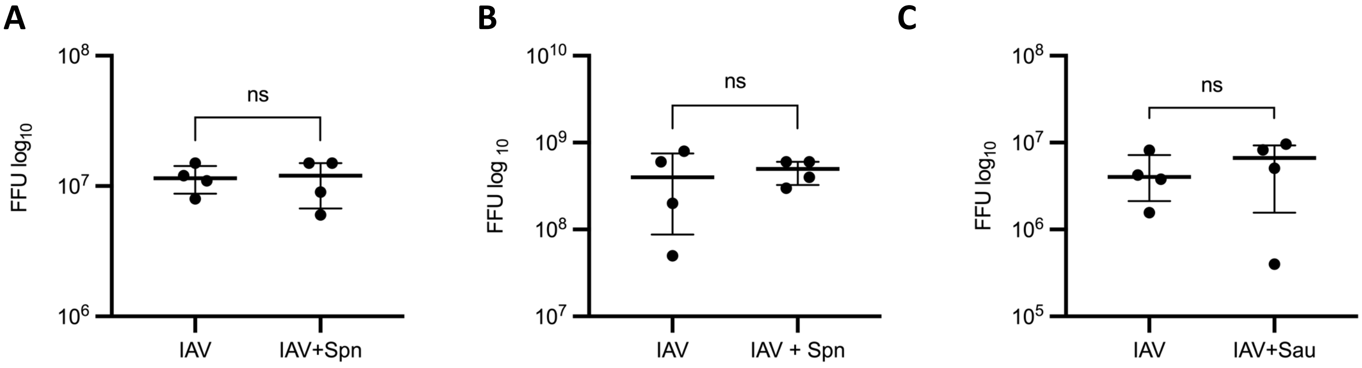

Supplemental Figure 2. (**A**) Primary differentiated human nasal cells or (**B**) HBECs isolated from the lungs of a 15 month old were infected with 100,000 PFU of IAV for 72 hours, followed by infection with 1,000 CFU *Spn.* IAV was quantified by foci forming assay of apical washes collected after six hours of *Spn* infection. (**C**) HBECs were infected with 100,000 PFU of IAV for 72 hours, followed by infection with 1,000 CFU *Staphylococcus aureus.* IAV was quantified by foci forming assay of apical washes collected after six hours of *Staphylococcus aureus* infection. All panels were analyzed by unpaired t-test. *, **, ***, **** indicates p of < 0.05, 0.01, 0.001, 0.0001 respectively. Brackets indicate median and interquartile range.

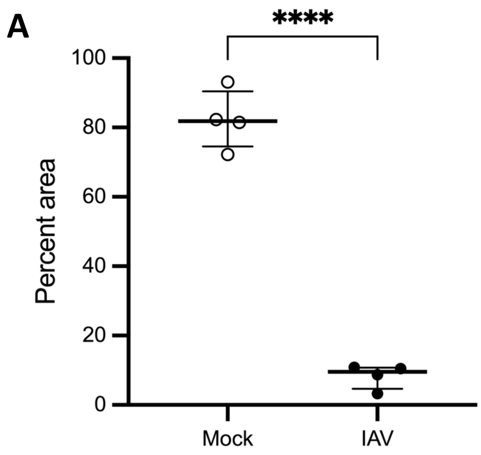

Supplemental Figure 3. (**A**) HBECs were infected with 100,000 PFU IAV for 72 hours and stained for cilia (white), F-actin (green), IAV (red) and mounted in DAPI Fluoromount (blue). Images were taken at 40X magnification on a Nikon A1R-HD25. Cilia was quantified using Image J on four fields of view from one biological replicate.

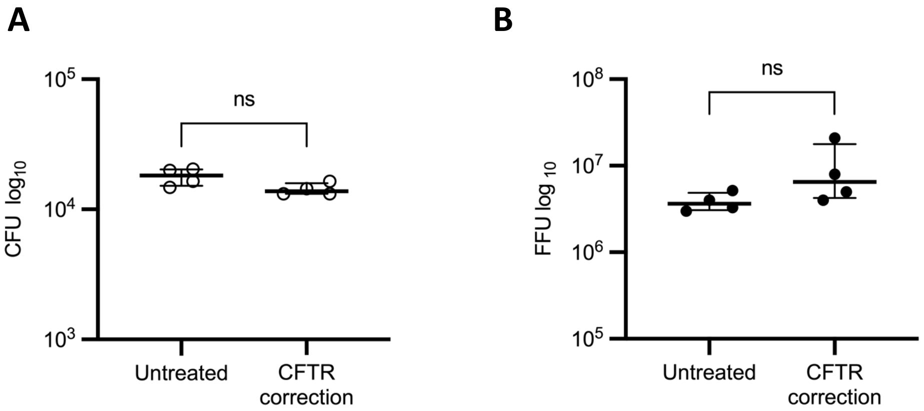

Supplemental Figure 4. (**A**) HBECs were basally treated with 10uM lumacaftor and tezacaftor for 72 hours and basally treated with 10uM ivacaftor overnight before *Spn* infection. After treatment, HBECs were infected with 1,000 CFU for six hours before apical washes were collected. *Spn* was quantified by vertical plating of apical washes. (**B**) HBECs were infected with IAV and basally treated with 10uM lumacaftor and tezacaftor and then basally treated with 10uM ivacaftor overnight before apical wash collection. After 72 hours of IAV infection, apical washes were collected and IAV was quantified by foci forming assay. All panels were analyzed by unpaired t-test. *, **, ***, **** indicates p of < 0.05, 0.01, 0.001, 0.0001 respectively. Brackets indicate median and interquartile range. Open circles indicate mock IAV infection, closed circles indicate IAV infection.

A

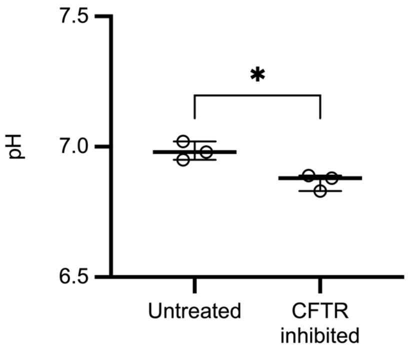

Supplemental Figure 5. (**A**) HBECs were basally treated with 20uM of CFTRinh172 for 72 hours before pH measurements were taken. Analyzed by unpaired t-test, * indicates $p<0.05$. Brackets indicate median and interquartile range.

Categories	Diseases or Functions Annotation	p-value	Activation z-score	Molecules	# Proteins in Data
Infectious Diseases, Organismal Injury and Abnormalities	Viral Infection	3.24E-20	-1.71	ACLY,ACTR2,ANK3,APOD,ARRDC1,ATP5F1B,ATP5IF1,B2M,BAIAP2,C3,C4A/C4B,C6,CAPN5,CCT2,CCT8,CD14,CD55,CDH1,CFB,CHMP4B,CIB1,CLIP1,CLTC,CNP,COPB1,COPB2,CROCC,CSE1L,CXCL10,DCTN1,DDR1,DDX42,DHX15,EEF2,EIF2S1,EIF3E,EIF3I,EPS8,F11R,FASN,FLII,FLOT2,FUBP1,GAPDH,HSPA4,HSPA5,ISG15,KARS1,LCN2,LMNA,LTF,MAT2A,MGAT5,MVP,MX1,MYO5B,MYOF,NCL,NPM1,NRP2,NT5E,PDCD6IP,PKM,PML,PPIB,PROS1,PRPF8,PSMA2,RAC1,RALB,RAP1B,RNH1,RPL12,RPL5,RPS10,RPS14,RPS16,RPS5,SERPINA1,SERPINE1,SLC9A3R1,SLPI,SNRPA,SNRPD1,SPINT1,SPON2,SPTBN1,SSB,ST6GAL1,STIP1,TMEM132A,TSG101,VCP,WFDC2,XPNPEP1,XPO1	96
Infectious Diseases,Organismal Injury and Abnormalities	Replication of virus	3.97E-10	-0.87	ATP5F1B,B2M,CHMP4B,COPB1,COPB2,CSE1L,CXCL10,DDX42,EIF2S1,EIF3E,F11R,FASN,FUBP1,HSPA5,ISG15,MVP,MX1,MYO5B,NCL,NPM1,PDCD6IP,PML,PRPF8,RPS10,RPS14,RPS16,RPS5,SERPINA1,SLC9A3R1,SLPI,SNRPD1,TSG101,VCP,XPNPEP1,XPO1	35
Infectious Diseases,Organismal Injury and Abnormalities	Sepsis	8.98E-10	-1.485	ACTR2,B2M,BAIAP2,C3,C4A/C4B,CD14,CD55,CLTC,COPB1,CTSC,EEF2,GOLM1,HSPA5,ISG15,LCN2,LMNB1,LTF,MUC1,MX1,RALB,RAP1B,SEC23A,SEC23B,SERPINE1,SERPING1,VAPA	26
Infectious Diseases,Organismal Injury and Abnormalities	Release of virus	8.7E-07	1.649	CHMP4B,HSPA5,ISG15,LMNA,PDCD6IP,SSB,TSG101,VCP	8
Infectious Diseases,Organismal Injury and Abnormalities	Production of virus	6.72E-06	-0.243	CCT8,CD14,CNP,CSE1L,MX1,PDCD6IP,PML,SERPINA1,TSG101,XPO1	10
Infectious Diseases,Organismal Injury and Abnormalities	Replication of Influenza A virus	1.62E-05	-2.034	B2M,COPB1,COPB2,CSE1L,DDX42,ISG15,MX1,PML,PRPF8,RPS10,RPS14,RPS16,RPS5,SLPI,XPNPEP1,XPO1	16
Cell-To-Cell Signaling and Interaction	Binding of bacteria	0.000015	1.757	CD55,CFH,LTF,MUC1,NCL,PIGR	6
Cell-To-Cell Signaling and Interaction	Adhesion of bacteria	2.94E-05	1.977	CFH,LTF,MUC1,PIGR	4
Cellular Function and Maintenance	Engulfment of bacteria	4.49E-05	2.604	C3,C6,CD14,CFH,LCN2,PIGR,RAC1	7
Humoral Immune Response,Inflammatory Response	Complement-dependent cytotoxicity	4.85E-05		C3,CD55,CD59,CFH	4
Organismal Injury and Abnormalities,Respiratory Disease	Lung injury	7.04E-05	0	AZGP1,C3,C4A/C4B,CD14,CFB,CXCL10,GAPDH,HSPA5,MMP7,MUC1,NT5E,PKM,RAC1,SERPINE1	14
Organismal Injury and Abnormalities,Respiratory Disease	Damage of lung	3.72E-06	-0.045	AZGP1,C3,C4A/C4B,CD14,CFB,CXCL10,GAPDH,HSPA5,MMP7,MUC1,MVP,NT5E,PKM,RAC1,SERPINE1,SERPING1,SLPI	17

Supplemental table 1. HBECs were infected with 100,000 PFU of IAV for 72 hours before apical washes were collected for proteomic analysis. GO analysis was conducted using the Qiagen IPA software following the standard statistical analysis protocol.
